# PReS-FINAL-2214: Validation of new pediatric criteria in diagnosis of Familial Mediterranean Fever in children

**DOI:** 10.1186/1546-0096-11-S2-P204

**Published:** 2013-12-05

**Authors:** E Baskin, US Bayrakci, N Akyol, K Gulleroglu, A Kantar, O Yurtseven, F Şahin

**Affiliations:** 1 Pediatric Nephrology and Rheumatology, Baskent University, Ankara, Turkey; 2 Pediatrics, Baskent University, Ankara, Turkey; 3 Genetics, Baskent University, Ankara, Turkey

## Introduction

Familial Mediterranean Fever (FMF) is the most common recurrent autoinflammatory fever syndrome. The diagnosis is based on clinical findings and supported by genetic analysis. Recently new set of diagnostic criteria were established for the diagnosis of familial Mediterranean fever (FMF) in childhood by Yalcinkaya et al.

## Objectives

The aim of this study was to validate these new criteria set among FMF patients and to compare it by Tel Hashomer criteria.

## Methods

Patient group was composed of 135 FMF patients. 165 patients who were admitted to our outpatient clinic with FMF like symptoms were reviewed as a control group. Demographic findings and laboratory examination of both groups were reviewed retrospectively. According to the new criteria, the diagnosis of FMF was established by the presence of two or more of five criteria (fever, abdominal pain, chest pain, arthritis, family history of FMF). Patients were evaluated by the new criteria set and also by the Tel Hashomer criteria and both criteria were compared in the terms of sensitivity and specificity.

## Results

Mean age of the patients was 14.02 ± 5.32 years (min-max: 1,1-18). Mean age of the symptoms was 6.98 ± 4.65 (min-max: 0.6-17) years. Mean age of diagnosis was detected as 8.76 ± 4.58 years (min-max:1.1-18). The mean time of diagnostic delay was detected as 1.8 years.

New sets of pediatric diagnostic criteria were found to be as specific and as sensitive as Tel Hashomer criteria. The 5 new criteria that was used by Yalcinkaya was found in patient with FMF in a significantly higher manner than the controls (p < 0.05). The presence of at least 2 criteria of Yalcinkaya's criteria was seen to be adequate to diagnose FMF. When we evaluated the relation between genetic features and new pediatric criteria we found that the specificity and sensitivity of these criteria was similar for patients with heterozygous mutations, compound heterozygous and patients with no mutation as well as homozygous patients.

## Conclusion

This study has suggested that sensitivity and specificity of the new criteria set is as high as Tel Hashomer criteria.

## Disclosure of interest

None declared.

